# Impact of HFOV in pARDS outcomes: questions remain

**DOI:** 10.1186/s13054-020-2837-3

**Published:** 2020-03-26

**Authors:** Hari Krishnan Kanthimathinathan, Martin C. J. Kneyber

**Affiliations:** 1 PICU, Birmingham Children’s Hospital, Steelhouse Lane, Birmingham, B4 6NH UK; 2 Department of Paediatrics, Division of Paediatric Critical Care Medicine, Beatrix Children’s Hospital, University Medical Center Groningen, Huispost CA 80, P.O. Box 30.001, 9700 RB Groningen, The Netherlands

We read with interest the analysis comparing outcomes in paediatric acute respiratory distress syndrome (pARDS) among those who required high-frequency oscillatory ventilation (HFOV) as ‘rescue therapy’, compared to those who did not [1]. The authors must be congratulated for using multiple matching techniques to account for some confounders. We also commend them for making the code used for analysis public [2]. We wish to clarify certain aspects of the study that may aid readers understand the context of the analysis better.

1. The dataset used for genetic matching included at least 68 ‘matched-pairs’ of severe pARDS. However, the 68 ‘matches’ were derived from a pool of less than 39 ‘real’ patients with severe pARDS in the non-HFOV cohort. While multiple matching with replacement may improve matching, it would be helpful to understand how many multiple matches were made from the smaller pool of non-HFOV patients and whether statistical adjustments were made for the duplicates in addition to a pairwise comparison.
2. The authors reported that the sensitivity analysis showed consistent findings with the primary analysis. Curiously, the odds ratios of mortality for mild, moderate and severe pARDS in the logistic regression analysis were 1.61, 1.02, and 1.00 respectively. This paradoxical trend may indicate an interaction between the use of HFOV and presence of moderate (or) severe pARDS, rather than the risk of HFOV per se. Was an interaction between the use of HFOV and severity of pARDS tested and accounted for in the logistic regression analysis?
3. The intention to match patients in a propensity- or genetic-matched study is to account for confounders present, when an intervention was indicated, between those that received the intervention compared to those that did not. The use of day 1 OI, rather than the OI immediately prior to the initiation of HFOV, may have systematically underestimated the true severity of pARDS in the HFOV group, given that HFOV was used as a ‘rescue mode’ anytime within 7 days. An equivalent assignment of pARDS severity could be done using the ‘highest OI’ within the first 7 days in the non-HFOV group. Was the use of OI prior to initiation of HFOV, rather than day 1 OI, considered for analysis?

## Authors’ response

Judith Ju Ming Wong, Siqi Liu, Mengling Feng, Jan Hau Lee

We thank Drs. Krishnan and Kneyber for their letter and the opportunity to clarify questions that remain.

1. In the genetic matching (GM) model, we used matching with replacement aiming to have a larger matched cohort [1]. Among the 67 uniquely matched non-high frequency oscillatory ventilation (HFOV) patients, majority were matched once [40/67 (59.7%)]. The remainder were matched twice [16/67 (23.9%)], thrice [5/67 (7.5%)] and more than three times [6/67 (9.0%)]. No adjustments were made for multiple matched pairs. However, experiments of GM without replacement showed consistent findings with our main analysis [odds ratio (OR) for mortality 1.9, 95% confidence interval (CI) 1.0 to 4.0; *p* = 0.09] lending confidence to the results.
2. We performed logistic regression with interaction term between HFOV use and paediatric acute respiratory distress syndrome (PARDS) severity and compared this with the original model without the interaction term using chi-square test and the ‘ANOVA’ function in R software. There was no improvement (*p* = 0.16) with introducing the interaction term, and hence, this was omitted.
  We hypothesized that HFOV use is beneficial in certain patient subgroups. We reported subgroup analyses (severe and non-severe PARDS) in the supplementary material (Table S3.2). A trend towards harm was associated with HFOV use in the non-severe [OR 2.9 (95% CI 1.1 to 8.1); *p* = 0.06] and severe [OR 1.6 (95% CI 0.7 to 3.5); *p* = 0.29] cohort, but the effect size in severe PARDS seemed lower. This paradoxical trend was similar to the multivariate analysis. However, this hypothesis may only be answered with the completion of the PROSPect trial (NCT03896763).
3. In our main analysis, we used oxygenation index (OI) calculated on the second day of PARDS, which has strong correlation to clinical outcomes [3]. However, anticipating the possibility of PARDS progression in the following days leading up to HFOV use, we also performed a sensitivity analysis using *daily OI* to adjust for time-varying confounding in the first week of PARDS (Tables S7.1 and S7.2). All three statistical approaches (GM, propensity score matching and marginal structural modelling) demonstrate a consistent direction of harmful effect. This approach is likely more robust than using a single day’s OI value.

In summary, our findings remain consistent—there is a signal toward harm in using HFOV in the general cohort of PARDS patients, especially in non-severe cases who otherwise have no other organ involvement. For the subgroup with severe PARDS, an empirical study is necessary and warranted to determine the effects of HFOV.

## Data Availability

Not applicable
